# ﻿Revisiting the phylogeny and taxonomy of the genus *Sidera* (Hymenochaetales, Basidiomycota) with particular emphasis on *S.vulgaris*

**DOI:** 10.3897/mycokeys.105.121601

**Published:** 2024-05-07

**Authors:** Vassiliki Fryssouli, Elias Polemis, Milton A. Typas, Georgios I. Zervakis

**Affiliations:** 1 Agricultural University of Athens, Laboratory of General and Agricultural Microbiology, Iera Odos 75, 11855 Athens, Greece Agricultural University of Athens Athens Greece; 2 National and Kapodistrian University of Athens, Department of Genetics and Biotechnology, Faculty of Biology, Panepistemiopolis, Athens 15701, Greece National and Kapodistrian University of Athens Athens Greece

**Keywords:** Basidiomycetes, biodiversity, fungal phylogeny, Mediterranean Europe, mushroom, white-rot fungi

## Abstract

The genus *Sidera* (Hymenochaetales, Basidiomycota) comprises white-rot, mono- or dimitic fungi with poroid or hydnoid hymenophore. It has a worldwide distribution albeit with fewer species present in the Southern Hemisphere. Although recent studies revealed the existence of several new *Sidera* species, there are still taxonomic inconsistencies and obscure phylogenetic relationships amongst certain taxa of the genus. In this work, a large number of *Sidera* collections were used to obtain an updated phylogeny, based on ITS and 28S rDNA sequences by including new material from Mediterranean Europe. The monophyly of the genus was strongly supported and all species with poroid hymenophore formed a highly-supported lineage with two major subclades. In total, 23 putative species were recognised. Amongst those, five are considered to possibly represent entities new to science, but further work is required since they are represented by single specimens or environmental sequences. Examined collections originally named *S.lenis* from southern Europe were grouped within *S.vulgaris*. Similarly, several collections under various names were hereby identified as *S.vulgaris*, including those of the recently described species *S.tibetica*. Furthermore, a critical discussion (based on morphoanatomical findings) is made on the key features that could be used to distinguish *S.lenis* from *S.vulgaris*.

## ﻿Introduction

The genus *Sidera* Miettinen & K.H. Larss. (Sideraceae, Hymenochaetales, Basidiomycota) was established to harbour four resupinate, wood-inhabiting, white-rot species, in accordance with morphological and phylogenetic evidence: *S.lenis* (P. Karst.) Miettinen (type species), *S.lowei* (Rajchenb.) Miettinen, *S.lunata* (Romell ex Bourdot & Galzin) K.H. Larsson and *S.vulgaris* (Fr.) Miettinen (Miettinen and Larsson 2011). Amongst them, *S.lowei* was originally described from Brazil (Rajchenberg 1987), while all the others were described from material collected in Europe. Recently, the diversity of the genus was significantly expanded as a result of phylogenetic studies using nuclear ribosomal markers (ITS and LSU) to describe new taxa from boreal, temperate and tropical habitats (Du et al. 2019, 2020; Liu et al. 2021, 2022, 2023; Xu et al. 2023).

Morphologically, *Sidera* species are characterised by resupinate, whitish to cream-coloured, yellowish or buff, rarely pinkish or bluish basidiomata, poroid hymenophore with middle-sized to small pores (and, in only one case, hydnoid hymenophore; i.e. *S.lunata*), monomitic or dimitic hyphal system, generative hyphae with clamps, rather loosely arranged skeletal hyphae, presence of rosette-like crystals in subiculum and/or trama, hymenial cystidia as thin-walled hyphidia (cystidioles) and minute, allantoid to lunate, hyaline, thin-walled, negative in Melzer’s reagent and acyanophilous basidiospores (Miettinen and Larsson 2011).

After re-examining the lectotype of *Physisporuslenis* (designated by Lowe 1956), Niemelä and Dai (1997) recognised two morphologically distinct species: one that was more common in boreal, mostly pine-inhabiting old-growth forests, i.e. *Skeletocutislenis* (P. Karst.) Niemelä and another species that came across more as generalist – in terms of substrate preference – and with a more southern distribution, i.e. *Sk.vulgaris* (Fr.) Niemelä & Y.C. Dai. In the original description of the genus *Sidera*, Miettinen and Larsson (2011) used two sequences which were then considered to represent *S.vulgaris*, both originating from Oceania (Tasmania, Gates FF257; New Zealand, Ryvarden 37198). However, no sequences from European collections were included in their phylogenetic analysis. The sequence from Tasmania nested close to *S.lowei*, while the sequence from New Zealand appeared as sister to *S.lenis*, indicating that they possibly correspond to two distinct species. Recent phylogenetic studies in the genus *Sidera* repeatedly used the aforementioned two specimens (Du et al. 2019, 2020) without including any *S.vulgaris* material from Europe. In fact, in one of these publications (Du et al. 2020), the specimen from Tasmania was identified as *S.minutipora* (Rodway & Cleland) Y.C. Dai, F. Wu, G.M. Gates & Rui Du. Moreover, Liu et al. (2021) used the term “*Sideravulgarissensu lato*” to refer to the New Zealand sequence and to some newly-generated sequences from Chinese collections, which were later linked to several new species, i.e. *S.americana* Z.B. Liu & Yuan Yuan, *S.borealis* Z.B. Liu & Yuan Yuan, and *S.tibetica* Z.B. Liu, J. Yu & F. Wu (Liu et al. 2022, 2023). Therefore, the phylogenetic position of *S.vulgaris* within the genus remains obscure and controversial.

In order to resolve this issue and provide an updated phylogeny of the genus *Sidera*, several collections from Mediterranean Europe, initially identified as *Skeletocutis* sp., *Sk.lenis* and *Sk.vulgaris* from Mediterranean Europe, were included in this study, together with a large number of publicly available sequences. In addition, answers were sought to the following key questions: (a) Could available specimens confirm the presence of *S.lenis* in the Mediterranean Region? (b) Is there adequate evidence that *S.vulgaris* indeed has a cosmopolitan distribution? (c) Which *Sidera* taxa are accommodated within *S.vulgarissensu lato* and are they related to new species correctly introduced in this genus? (d) What are the key morphological features to distinguish *S.vulgaris* from *S.lenis*?

## ﻿Methods

### ﻿Biological material – Morphology

Voucher specimens studied were deposited in the fungaria of the Laboratory of General and Agricultural Microbiology (Agricultural University of Athens, ACAM), the University of Oslo (O, HUBO) and the University of Salamanca (Salamanca, SALA-Fungi). Pore density was studied using a stereomicroscope (Zeiss Stemi 2000‐C) at 10–20× magnification by measuring the number of pores per mm. Microscopic examination was performed with a Zeiss AxioImager A2 microscope under bright field and differential interference contrast (DIC) and microphotographs were taken with the aid of a mounted digital camera (Axiocam). Examination of microscopic features were performed in Cotton Blue, Melzer’s reagent and 5% potassium hydroxide (KOH) mounting media. Measurements were taken in KOH under 1,000× magnification and DIC. For each specimen studied, a minimum number of 25 basidiospores were measured and their size (with standard deviation, SD) is provided as minimum and maximum average (average – SD and average + SD, respectively). In addition, the quotient (Q) for each basidiospore was calculated and is presented together with the respective average values (Qav). Length and width of basidia and hymenial cystidioles are also presented with the same formula: (n = x/y) refers to x measurements (of pores/mm, basidiospores, basidia and cystidioles) from y specimens. Other essential microscopical features which were also examined, including generative and skeletal hyphae from subiculum and hymenophoral trama (tubes) and the presence of stellate crystals and capitate hyphal tips.

### ﻿DNA extraction, amplification and sequencing

Total genomic DNA was extracted from dried material using the Nucleospin Plant II kit (Macherey and Nagel, USA) according to manufacturer’s protocol with minor modifications (Zervakis et al. 2019). The two regions of the nuclear ribosomal repeat unit – namely the ITS region and a fragment of the ribosomal large subunit gene (28S/LSU) – were amplified through the polymerase chain reaction (PCR) in a MiniAmp Plus Thermal Cycler (Applied Biosystems, CA, USA). The ITS sequences were generated using the forward and backward primers ITS1 and ITS4 (White et al. 1990) to include partial 18S, complete ITS and partial 28S rDNA. The 28S rDNA sequences included the D1/D2 domain by employing the primers LROR and LR5 (Vilgalys and Hester 1990; Stielow et al. 2015). The PCR procedure for ITS was as follows: initial denaturation at 95 °C for 3 min followed by 35 cycles at 94 °C for 40 sec, 52 °C for 45 sec and 72 °C for 1 min and a final extension step of 72 °C for 10 min. The PCR procedure for 28S rDNA was as follows: initial denaturation at 94 °C for 3 min, followed by 35 cycles at 94 °C for 30 sec, 48 °C for 1 min and 72 °C for 1.5 min and a final extension of 72 °C for 10 min. The PCR products were purified with Pure Clean spin columns (Invitrogen, California, USA) following the manufacturer’s instructions.

The PCR products were sequenced using the same forward and backward primers with the amplification procedure in an automated ABI sequencer (Life Technology) at CeMIA Inc. (Larissa, Greece). Trace files obtained from the sequencer were aligned using MEGA 11 (Tamura et al. 2021). Consensus sequences were manually edited to remove or replace all ambiguous characters and were cross-checked against local and public databases, including the international nucleotide sequence database collaboration (INSDC, Arita et al. (2021)) and UNITE (Nilsson et al. 2019). Validated sequences were submitted to GenBank (Sayers et al. 2024) to obtain accession numbers (Table 1).

**Table 1.** Biological material used in the phylogenetic analysis of genus *Sidera*. Information includes final identification of taxa (as derived from the present study; in bold typeface), initial identification of taxa as submitted in public databases or as it appeared on the material examined here for first time (when another name appears in parenthesis, it corresponds to the one subsequently used when this collection served as type material), specimen code, geographic origin, substrate and corresponding GenBank accession numbers for ITS and 28S rDNA. The reference for each entry is also provided; asterisk (*) indicates those not accompanied by a publication. Collections serving as type material are indicated with superscript letters, i.e., ^H^: holotype, ^L^: lectotype, and ^P^: paratype; ‘n.a.’ denotes not available information.

Species	Collection code	Geographic origin	Substrate	GenBank accession no.		Reference
				ITS	28S rDNA	
***S.americana***						
*Sidera* sp. (*S.americana*)	Dai 12730^H^	USA: CT	on rotten stump of *Pinus*	MW198478	n.a.	Liu et al. (2021, 2023)
*S.malaysiana*	Dai 19173	Canada	on rotten angiosperm wood	MW198477	MW192005	Liu et al. (2022, 2023)
*Sidera* sp.	TUF101553	Estonia	*Pinussylvestris*	UDB015767	n.a.	Runnel (2010)*
*S.vulgaris*	Alden Dirks: ACD0413	USA: MI	n.a.	OL756000	OL742443	Dirks (2021)*
***S.borealis***						
*Sidera* sp. (*S.borealis*)	Cui 11216^H^	China: SN	fallen angiosperm trunk	MW198485	n.a.	Liu et al. (2021, 2023)
S.cf.vulgaris	Dai 22822	China: YN	on rotten wood of *Picea*	OM974254	OM974246	Liu et al. (2022, 2023)
*Sidera* sp.	TUF122801	Estonia	*Pinussylvestris*	UDB023006	UDB023006	Runnel (2013)*
***S.inflata***						
*S.inflata*	Cui 13610^H^	China: HI	on rotten angiosperm wood	MW198480	n.a.	Liu et al. (2021)
***S.lenis***						
*S.lenis*	O. Miettinen 11036.1^L^	Finland	n.a.	FN907914	FN907914	Miettinen and Larsson (2011)
*S.lenis*	NSK 1017015	Russia	n.a.	OR364533	n.a.	Vlasenko (2023)*
*S.lenis*	Dai 22834	China: YN	on rotten wood of *Picea*	OQ134538	n.a.	Liu et al. (2023)
*S.lenis*	TUF111091	Sweden	*Pinussylvestris*	UDB032409	n.a.	Sell (2015)*
***S.lowei***						
*S.lowei*	Dollinger 922	USA: FL	*Quercus*	KY264044	n.a.	Dollinger and Vlasak (2016)*
*S.lowei*	Ryvarden 40576	Venezuela	n.a.	FN907917	FN907917	Miettinen and Larsson (2011)
***S.lunata***						
*Athelopsislunata*	JS 15063 (1717)	Norway	n.a.	DQ873593	DQ873593	Larsson et al. (2006)
*S.lunata*	S851	Estonia	soil	UDB0662815	n.a.	Tedersoo et al. (2018)*
***S.malaysiana***						
*S.malaysiana*	Dai 18570^H^	Malaysia	on rotten angiosperm wood	MW198481	MW192007	Liu et al. (2021)
***S.minutipora***						
*S.minutipora*	Cui 16720	Australia: Tasmania	on rotten stump of *Eucalyptus*	MN621349	MN621348	Du et al. (2020)
*S.vulgaris*	G. Gates FF257	Australia: Tasmania	n.a.	FN907922	FN907922	Miettinen and Larsson (2011)
***S.minutissima***						
*S.minutissima*	Dai 19529 ^H^	Sri Lanka	on rotten angiosperm branch	MN621352	MN621350	Du et al. (2020)
*S.minutissima*	Dai 22495	China	n.a.	OM974248	OM974240	Liu et al. (2022)
*Sidera* sp.	KAS: L1620	Réunion Island	n.a.	UDB024833	n.a.	Ordynets (2015)*
*Sidera* sp.	TUF123971	Seychelles	n.a.	UDB039740	n.a.	Kõljalg (2018)*
***S.parallela***						
*S.parallela*	Cui 10346^H^	China: YN	on rotten angiosperm trunk	MK346145	n.a.	Du et al. (2020)
*S.parallela*	Cui 10361^P^	China: YN	on fallen angiosperm trunk	MK346144	n.a.	Du et al. (2020)
*S.parallela*	Dai 22038	China	n.a.	MW477793	MW474964	Liu et al. (2022)
***S.punctata***						
*S.punctata*	Dai 22119^H^	China: HI	on rotten angiosperm wood	MW418438	MW418437	Liu et al. (2021)
unc. fungus	L042880-122-060-A02	Ocean	air filter sample	GQ999131	n.a.	Fröhlich-Nowoisky et al. (2012)
unc. fungus	L042881-122-061-B08	Taiwan	air filter sample	GQ999432	n.a.	Fröhlich-Nowoisky et al. (2012)
***S.roseo-bubalina***						
*S.roseo-bubalina*	Dai 11277^T^	China: HA	under decay *Quercus*	MW198483	n.a.	Liu et al. (2021)
***S.salmonea***						
*S.salmonea*	Dai 23354^P^	China: Tibet	*Abies*	OM974250	OM974242	Liu et al. (2022)
*S.salmonea*	Dai 23428	China: Tibet	*Pinusarmandii*	OM974251	OM974243	Liu et al. (2022)
***S.srilankensis***						
*S.srilankensis*	Dai 19654^H^	Sri Lanka	on rotten angiosperm wood	MN621344	MN621346	Du et al. (2020)
*S.srilankensis*	Dai 19581^P^	Sri Lanka	on rotten angiosperm wood	MN621345	MN621347	Du et al. (2020)
***S.tenuis***						
*S.tenuis*	Dai 18697^H^	Australia: Tasmania	on rotten stump of *Eucalyptus*	MK331865	MK331867	Du et al. (2020)
*S.tenuis*	Dai 18698^P^	Australia	on rotten stump of *Eucalyptus*	MK331866	MK331868	Du et al. (2020)
***S.tianshanensis***						
*S.tianshanensis*	Cui 19143^H^	China: XJ	on fallen trunk of *Piceaschrenkiana*	OP920995	OP920987	Xu et al. (2023)
*S.tianshanensis*	Cui 19132	China: XJ	on stump of *Piceaschrenkiana*	OP920994	OP920986	Xu et al. (2023)
***S.vesiculosa***						
*S.vesiculosa*	BJFC025377^T^	Singapore	on rotten angiosperm	MH636564	MH636566	Du et al. (2019)
*S.vesiculosa*	BJFC025367^P^	Singapore	on rotten angiosperm	MH636565	MH636567	Du et al. (2019)
*Sidera* sp.	TUE002764	Papua New Guinea	soil	UDB07018609	n.a.	Tedersoo et al. (2020)*
***S.vulgaris***						
*S.vulgaris*	ACAM 2013-0017	Greece	*Pinushalepensis*	PP275215	PP275225	present work
*S.vulgaris*	ACAM DD2559	Greece	*Abiescephalonica*	PP275216	PP275226	present work
*Skeletocutisvulgaris*	HUBO 7745	Italy	*Pinussylvestris*	PP275217	PP275227	present work
*Skeletocutislenis*	HUBO 8296	Italy	*Fagus*	PP275218	PP275228	present work
*Skeletocutisvulgaris*	HUBO 8465	Italy	Pinusnigrassp.laricio	PP275219	PP275229	present work
*Skeletocutislenis*	SALA-Fungi 3749	Spain	*Eucalyptuscamaldulensis*	PP275220	n.a.	present work
*Skeletocutislenis*	SALA-Fungi 3752	Spain	*Pinuspinaster*	PP275221	n.a.	present work
*Skeletocutis* sp.	SALA-Fungi 4105	Spain	*Pinuspinaster*	PP275222	n.a.	present work
*Skeletocutis* sp.	SALA-Fungi 4111	Spain	*Acermonspessulatum*	PP275223	n.a.	present work
*S.vulgaris*	TU114503	Estonia	*Populustremula*	UDB034888	n.a.	Sell (2017)*
*S.vulgaris*	TU135349	Estonia	*Piceaabies*	UDB0754207	n.a.	Sell (2018)*
*Sidera* sp. (*S.tibetica*)	Dai 23648^H^	China: Tibet	*Pinusarmandii*	OM974253	OM974245	Liu et al. (2022)
*Sidera* sp. (*S.tibetica*)	Dai 23407^P^	China: Tibet	n.a.	OM974252	OM974244	Liu et al. (2022)
*S.tibetica*	Dai 22151	China	n.a.	MW477794	MW477794	Liu et al. (2021)
*S.tibetica*	Dai 21057	Belarus	on rotten wood of *Picea*	MW198484	MW192009	Liu et al. (2021)
*S.tibetica*	LE F-342597	Russia	Pinusbrutiavar.eldarica	OR457651	n.a.	Volobuev (2023)
*Schizopora* sp.	206	Spain	*Castaneasativa* EM root tips	MN947225	n.a.	Santolamazza-Carbone (2020)*
*Schizopora* sp.	DLL2009-014	USA: MN	*Populus* spp.	JQ673191	n.a.	Brazee et al. (2012)
*Schizopora* sp.	FH:BHI-F453	USA: MA	n.a.	MF161274	n.a.	Haelewaters et al. (2018)
*Sidera* sp.	UC2022907	USA: CA	on litter or well decayed wood in pinaceous forest	KP814250	n.a.	Rosenthal et al. (2017)
unc. *Hyphodontia*	1Bart548S	USA: NH	n.a.	HQ022192	n.a.	Vineis (2011)
unc. fungus	S38	Germany	air sample	FJ820526	n.a.	Fröhlich-Nowoisky et al. (2012)
***Sidera* sp. 1**						
*S.vulgaris*	Ryvarden 37198	New Zealand	n.a.	FN907918	FN907918	Miettinen and Larsson (2011)
***Sidera* sp. 2**						
*Sidera* sp.	UC2023008	USA: MS	decayed wood in pinaceous forest	KP814157	n.a.	Rosenthal et al. (2017)
***Sidera* sp. 3**						
*S.lowei*	Ryvarden 38817	New Zealand	n.a.	FN907919	FN907919	Miettinen and Larsson (2011)
***Sidera* sp. 4**						
unc. fungus	L042886-122-066-F04	Taiwan	air filter	GQ999509	n.a.	Fröhlich-Nowoisky et al. (2012)
unc. fungus	L042881-122-061-B09	Taiwan	air filter	GQ999433	n.a.	Fröhlich-Nowoisky et al. (2012)
***Sidera* sp. 5**						
*Sidera* sp.	MEL:2382752	Australia: NT	n.a.	n.a.	KP012935	Bonito et al. (2014)*
**Outgroups**						
*Alloclavariapurpurea*	Miettinen 18831	ΗΠΑ: WA	old-growth forest with conifers	ON188807	ON188807	Viner (2022)*
*Rickenellamellea*	Lamoure 74	n.a.	n.a.	U66438	U66438	Lutzoni (1997)
*Resiniciumfurfuraceum* (*Skvortzoviafurfuracea*)	KHL 11738	Finland	n.a.	DQ873648	DQ873648	Larsson et al. (2006)
*Skvortzoviafurfurella*	KHL 10180	Puerto Rico	n.a.	DQ873649	DQ873649	Larsson et al. (2006)

### ﻿Phylogenetic analysis

Phylogenetic analysis of the genus *Sidera* was performed using Maximum Likelihood (ML) and Bayesian Inference (BI) approaches with separate ITS and 28S rDNA datasets. In addition, a concatenated two-marker dataset was analysed by including specimens with data available for both markers. Besides the newly-generated sequences, additional reference sequences were retrieved from INSDC and UNITE using BLASTn searches. *Skvortzoviafurfurella* (Bres.) Bononi & Hjortstam and *Skvortzoviafurfuracea* (Bres.) G. Gruhn & Hallenberg were used as outgroups (Liu et al. 2022), while two additional Hymenochaetales species, i.e. *Alloclavariapurpurea* (O.F. Müll.) Dentinger & D.J. McLaughlin and *Rickenellamellea* (Singer & Clémençon) Lamoure, were also included in the analysis. A detailed list of specimens examined in the present study, along with corresponding information, for example, initial identification, habitat, sequence database accession number and pertinent publication (when available), is provided in Table 1.

Sequences were aligned by the online version of MAFFT v. 7 (Katoh et al. 2019) using the “G-INS-i” and “Q-INS-I” strategies for ITS for 28S rDNA barcodes, respectively. The alignments were visually inspected and manually adjusted for conspicuous errors and gapped sites in Mesquite 3.81 (Maddison and Maddison 2023). The MUMSA tool (Lassmann and Sonnhammer 2006) was used to select the best alignment.

ML analysis was performed using IQ-TREE v. 2.2.7 (Minh et al. 2022) via the CIPRES Science Gateway (Miller et al. 2015) with a random starting tree. All model parameters were estimated by the software. The best Maximum Likelihood tree was retained from all searches and the Maximum Likelihood bootstrap values (ML-BS) were determined using ultrafast bootstrapping algorithm with 10,000 replicates. BI analysis was implemented in MrBayes v.3.2.7a (Ronquist et al. 2012) employing optimal models of evolution determined for ITS and 28S rDNA with jModelTest2 v.2.1.6 (Darriba et al. 2012) under the Bayesian Information Criterion (BIC). Two parallel runs, each one consisting of four incrementally heated Monte Carlo Markov Chains, were initiated from programme-generated random trees. The analysis involved sampling every 1,000^th^ generation until the average standard deviation of split frequency fell below 0.005. The burn-in phase (first 25% of sampled trees) was discarded. The remaining trees were used to generate a 50% majority rule consensus tree and to estimate the Bayesian posterior probabilities (BPPs). Branches with ML-BS and BPPs equal to or above 65% and 0.95, respectively, were considered as significantly supported.

The best topologies from MP analyses are presented and the final alignments and the phylograms are deposited in TreeBASE (http://www.treebase.org) under accession ID: 31153. The sequence identity was calculated using MAFFT, accessed through EMBL-EBI (https://www.ebi.ac.uk/Tools/msa/mafft/).

## ﻿Results

### ﻿Taxonomy – Morphology

#### ﻿ Sidera vulgaris

Taxon classificationFungiHymenochaetalesCantharellaceae

(Fr.) Miettinen, Mycological Progress 10 (2): 136 (2011)

457C3BB2-2DC5-5877-A377-AF3D8B92B624

Figs 1 , 2

Polyporus vulgaris Fr., Systema Mycologicum 1: 381 (1821). Basionym.
Skeletocutis vulgaris (Fr.) Niemelä & Y.C. Dai, Annales Botanici Fennici 34 (2): 135 (1997). Synonyms.

##### Description.

***Basidioma***—Annual to biennial, resupinate, soft when fresh and rather tough, soft-corky after drying, confluent and widely effused covering extended under-surface of decaying logs, 0.8–2.0 mm thick at the centre; pore surface white to cream when fresh, becoming yellowish to buff when dry; sterile margin indistinct, cottony, white, thinning-out; pores very small, roundish, (5) 6–8 (10) per mm (n = 273/13); dissepiments thin, entire to slightly lacerate; subiculum very thin, cottony, concolorous with the tube layer; tubes concolorous with the poroid surface, up to 2 mm long.

**Figure 1.**
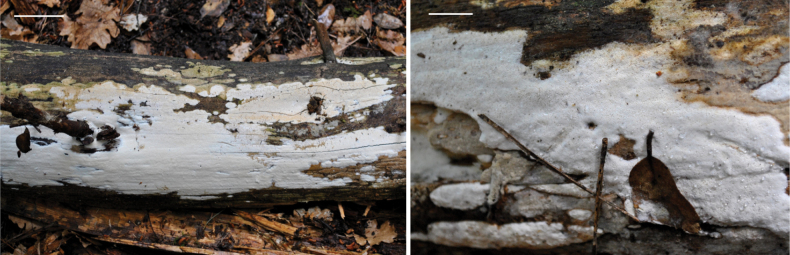
*S.vulgaris* specimen *in situ* (ΑCAM 2013-0017). Scale bar: 5 cm (left); 2 cm (right).

Hyphal system dimitic in all parts of the basidioma; generative hyphae smooth, without encrustations, septa with clamp connections; skeletal hyphae not reacting with Cotton Blue, Melzer’s reagent or KOH.

***Subiculum***—Hyphae interwoven, skeletal hyphae dominating, skeletals (1.7) 2–3.5 (4.0) μm in diameter, rosette-like crystal clusters rare to common.

***Tubes***—Hyphae subparallel to moderately interwoven. Generative hyphae, thin to slightly thick-walled, poorly branched, 1.7–3.0 μm in diameter. Skeletal hyphae, thick-walled to subsolid, hyaline, rarely branched, flexuous, 1.7–3.5 μm in diameter, with scattered swellings up to 7 μm. Dissepiment edges with both generative and skeletal hyphae that often bear a swollen, capitate apex, generative hyphae sometimes covered by a mucous droplet, rosette-like crystals frequent in mature basidiomata. Cystidioles seldom to abundant, fusoid, thin-walled, hyaline, basally swollen, with hyphoid neck and mostly obtuse or capitate tip, some bearing crystals at apex (asterocystidia), a few modified as halocystidia were also observed, (9.3) 12.4–19.9 (25.0) × (2.2) 2.8–4.0 (5.3) μm (n = 125/15). Basidia barrel-shaped to somewhat short-clavate, with four sterigmata and a basal clamp, (6.2) 6.8–9.9 (14.6) × (3.1) 3.8–4.7 (5.6) μm (n = 185/15); basidioles barrel-shaped, slightly shorter than the basidia.

**Figure 2.**
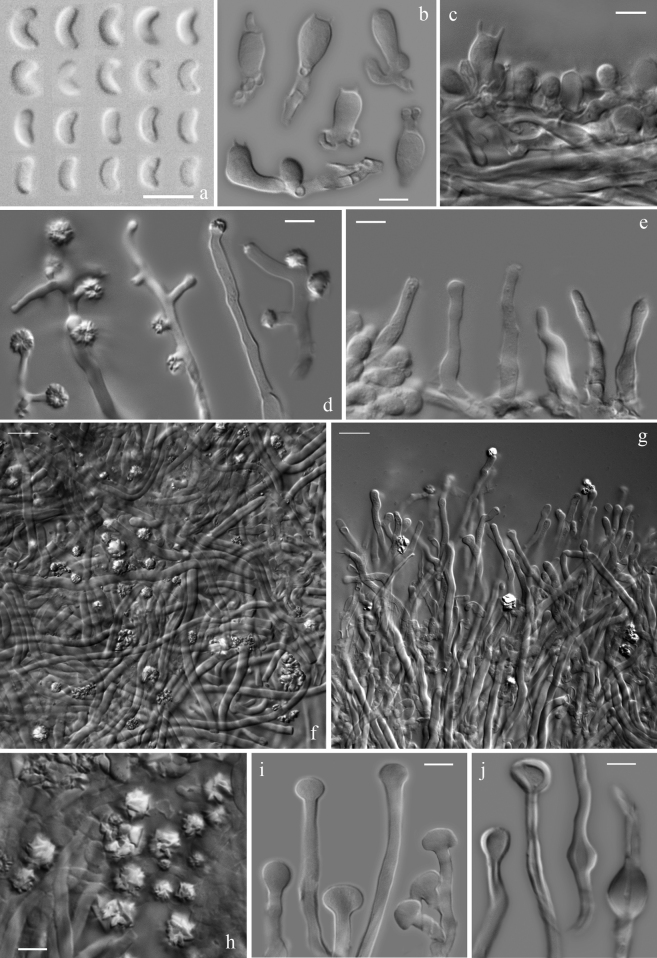
Micromorphological features of *S.vulgaris*; scale bar 5 μm [except of **f** and **g** 10 μm] **a** basidiospores (all specimens) **b** basidia (ACAM 2013-0017, ACAM DD2559, HUBO 7745, HUBO 8296, HUBO 8465, SALA-Fungi 3752) **c** hymenium with basidia and basidioles (HUBO 8465) **d** branched and unbranched cystidioles bearing crystals at apex (asterocystidia) (ACAM DD2559, SALA-Fungi 3752, SALA-Fungi 4111) **e** hymenial cystidioles (ACAM DD2559, HUBO 8296, SALA-Fungi 3749) **f** hyphae of the subiculum with dominating skeletals (ACAM 2013-0017) **g** dissepiment edges with skeletal and generative hyphal ends (HUBO 7745) **h** details of the rosette-like crystal clusters from tramal hyphae (HUBO 7745) **i** capitate ends of generative hyphae from dissepiments and hymenium (with mucous droplets) (HUBO 8296, SALA-Fungi 3749, SALA-Fungi 3752) **j** skeletal hyphae from dissepiments with swellings (ACAM 2013-0017, HUBO 7745, SALA-Fungi 3752, SALA-Fungi 4111).

***Basidiospores***—Cylindrical, moderately curved to lunate, thin-walled, hyaline, smooth, negative in Melzer’s reagent, acyanophilous, (3.0) 3.4–3.9 (4.3) × (1.2) 1.4–1.6 (1.8) μm, Average = 3.6 × 1.5 μm, Q = (1.95) 2.24–2.60 (3.08) Q_AV_ = 2.41 (n = 399/15).

##### Distribution and hosts.

The species is reported from Mediterranean Europe (e.g. Portugal, Spain, France, Italy, Croatia and Greece), Germany, Slovakia, Poland, Estonia, Sweden, Belarus, Russia, as well as from Armenia, Georgia, Iran, Kazakhstan, China, USA and Canada (Niemelä and Dai 1997; Ghobad-Nejhad 2011; Bernicchia et al. 2020; Liu et al. 2022; this work). It occurs on various broadleaved trees of (*Alnus*, *Eucalyptus*, *Fagus*, *Populus*, *Quercus*, *Sorbus* and *Ulmus*), as well as on coniferous trees, i.e. *Picea*, *Pinus* (*P.halepensis*, P.nigrassp.laricio, *P.pinaster*, *P.sylvestris*) or *Juniperus* and on *Abiescephalonica* (this work).

##### Specimens examined.

Greece: Sterea Ellas, Fthiotida, Gardiki, on trunk of *Abiescephalonica*, 28 April 2007, ACAM DD2559, coll. D. Dimou. Attica, Mt. Parnitha, on trunk of *P.halepensis*, 30 May 2013, ACAM 2013-0017. coll. E. Polemis. ITALY: Emilia Romana, Forli, Pian del Pero Cullacea, on *Ulmusglabra*, 7 October 2002, HUBO 7629, coll. A. Bernicchia (as *Sk.vulgaris*); ibidem. on *Fagus* sp. 11 October 2006, HUBO 8296, coll. A. Bernicchia (as *Sk.lenis*); Ferrara, Bosco della Mesola, on *Populus* sp. 12 November 2003, HUBO 7701, coll. A. Bernicchia (as *Sk.lenis*); Bologna, Parko la Martina, on *P.sylvestris*, 16 July 2003, HUBO 7745, coll. A. Bernicchia (as *Sk.vulgaris*); Ravena, Pineta San Vitale, on *Populusalba*, 4 November 2003, HUBO 7811, coll./det. A. Bernicchia (as *Sk.vulgaris*). Sardinia, Tonara, Isca de sa Mela, on P.nigrassp.laricio, 14 October 2007, HUBO 8465, coll. L. Arras (as *Sk.vulgaris*); Sorgono, Isca de sa Mela, on P.nigrassp.laricio, 18 November 2009, HUBO 8522, coll. A. Bernicchia (as *Sk.lenis*). SPAIN: Castile-Leon, Garcibuey, on *Eucalyptuscamaldulensis*, 7 November 2005, SALA-Fungi 3749, ibidem. on *P.pinaster*, 22 November 2006, SALA-Fungi 3747, coll. S.P. Gorjón (as *Sk.lenis*); Herguijuela de la Sierra, on *P.pinaster*, 18 November 2007, SALA-Fungi 3752, coll. S.P. Gorjón (as *Sk.lenis*); Miranda del Castañar, on *P.pinaster*, 22 November 2006, SALA-Fungi 4105, coll. S.P. Gorjón (as *Skeletocutis* sp.); San Martín del Castañar, on *Acermonspessulatum*, 14 October 2007, SALA-Fungi 4111, coll. S.P. Gorjón (as *Skeletocutis* sp.); Cepeda, on *Alnusglutinosa*, 29 November 2006, SALA-Fungi 3745, coll. S.P. Gorjón (as *Sk.lenis*).

### ﻿Phylogenetic analysis

To estimate the phylogeny of the genus *Sidera*, datasets of ITS and 28S rDNA sequences were compiled, including sequences from collections with a Mediterranean distribution, as well as from pertinent specimens and environmental samples deposited in INSDC and UNITE in order to cover as much as possible the diversity and distribution of the genus. The total dataset consisted of 69 collections represented by 68 ITS and 36 28S sequences (Table 1). The material examined for the first time in the present study included nine collections from Mediterranean Europe, from which nine ITS and five 28S sequences were obtained. Additional information on the phylogenetic analyses performed for each dataset is provided in Suppl. material 1. Both applied phylogenetic strategies, ML and BI, produced phylograms characterised by a consistent topology, devoid of any supported conflicts.

**Figure 3.**
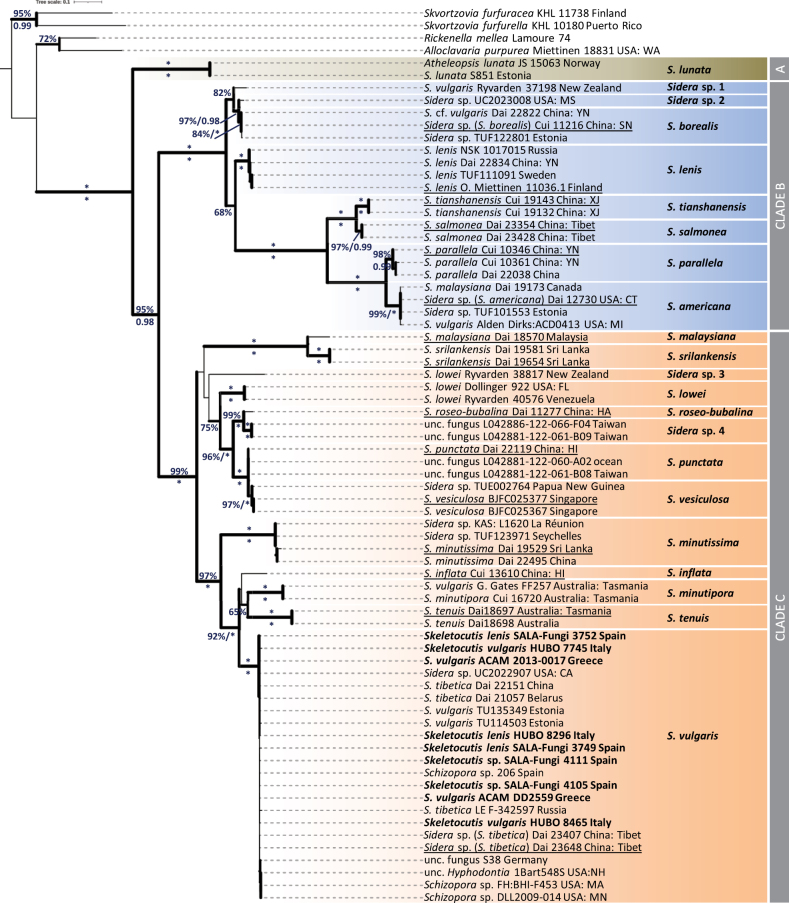
Phylogenetic relationships within the genus *Sidera* inferred by using ML analysis on the ITS sequence dataset. ML BS ≥ 65% and BPP ≥ 0.95 are appended to nodes; asterisk denotes 100% ML BS and/or 1.00 BBP. Specimens studied are followed by their voucher code and geographic origin. Sequences determined in the present study appear in bold, while those representing type material are underlined. The phylogram is rooted with *Skvortzoviafurfuracea* and *Skvortzoviafurfurella*. The scale bar indicates 0.1 expected change per site per branch.

The phylogenetic reconstruction, based on the ITS sequences (Fig. 3), recovered *Sidera* as a strongly-supported monophyletic clade (ML-BS 100%, BPP 1.00), which is further segregated into three well-supported main clades, A through to C. In total, 22 highly-supported terminal clades were recovered including those corresponding to the 18 formally described taxa; amongst them, 14 are represented by type sequences (plus one representing *S.tibetica*, which, however, should be considered as synonym of *S.vulgaris* as explained below). No sequences from type specimens were available for *S.lunata*, *S.vulgaris*, *S.lowei* or *S.minutipora*. In addition, four terminal clades do not correspond to the already known taxa and they could represent undescribed species. They are provisionally referred to as ‘*Sidera* sp. 1, 2, 3 and 4’.

Clade A (100%, 1.00) includes only *S.lunata*, which is distantly related to the rest of the *Sidera* spp. and it has a hydnoid hymenophore. All other taxa have poroid hymenophores and are grouped with significant support (95%, 0.98). They are further subdivided into clades B (100%, 1.00) and C (99%, 1.00) consisting of eight and 13 species, respectively. Clade B includes *S.lenis* – the type species of the genus – represented by collections from Sweden, Finland, Russia and China, as well as a cluster composed of *S.borealis* and two closely-related taxonomic entities. The first of them is hereby designated ‘*Sidera* sp. 1’ (UNITE DOI: SH1110196.09FU); it corresponds to the specimen Ryvarden 37198 from New Zealand, initially identified as *S.vulgaris*, but apparently not related to the real *S.vulgaris*, which is grouped in Clade C and includes material from the Northern Hemisphere. The second is represented by the specimen *Sidera* sp. UC2023008 from USA. Although closely positioned to *S.borealis*, it is considered as distinct from the latter species since it shows a rather low ITS sequence identity (96.5–98.4%) and distant geographic occurrence (*S.borealis* is reported from Europe and China). We provisionally call it ‘*Sidera* sp. 2’ (UNITE DOI: SH1110192.09FU). Moreover, clade B includes two pairs of sister species (100%, 1.00), i.e. *S.tianshanensis* B.K. Cui & T.M. Xu and *S.salmonea* Z.B. Liu, Jian Yu & F. Wu (both from Asia), as well as *S.parallela* Y.C. Dai, F. Wu, G.M. Gates & Rui Du and *S.americana* (the former originates from Asia, while the latter from North America and north Europe).

Clade C comprises the main diversity of the genus by accommodating 11 species and two entities possibly corresponding to new taxa. *S.srilankensis* Y.C. Dai, F. Wu, G.M. Gates & Rui Du and *S.malaysiana* Z.B. Liu & Y.C. Dai form a robustly-supported clade (100%, 1.00) consisting of Asian specimens. Similarly, sequences deriving from the Neotropics correspond to *S.lowei*. However, another collection (Ryvarden 38817) – initially identified as *S.lowei* from New Zealand – is phylogenetically separated from the previous species and seems to represent a distinct taxon (ITS sequence identity: 83.0–83.8%), herein called ‘*Sidera* sp. 3’ (UNITE DOI: SH1110192.09FU). Furthermore, *S.roseobubalina* Z.B. Liu & Y.C. Dai is represented only by the holotype, originating from China. It is related to two sequences derived from environmental samples (air filters, Taiwan; ITS sequence identity to *S.roseobubalina*: 93.5–93.6%); hence, the latter could possibly correspond to an undescribed taxon which is provisionally named ‘*Sidera* sp. 4’ (UNITE DOI: SH1111516.09FU). The aforementioned taxa are strongly linked (96%, 1.00) with a group consisting of *S.punctata* Z.B. Liu & Y.C. Dai and *S.vesiculosa* Rui Du & M. Zhou; these four species are represented by sequences from material of Asian origin. Finally, a well-supported cluster (97%, 1.00) is composed by *S.minutissima* Y.C. Dai, F. Wu, G.M. Gates & Rui Du (including specimens from islands of the Indian Ocean and China), *S.inflata* Z.B. Liu & Y.C. Dai from China (sequence data available only from the type collection), the sister species *S.minutipora* (Rodway & Cleland) Y.C. Dai, F. Wu, G.M. Gates & Rui Du and *S.tenuis* Y.C. Dai, F. Wu, G.M. Gates & Rui Du (consisting of material from Australia) and *S.vulgaris*.

*S.vulgaris* forms a highly-supported terminal clade (100%, 1.00) composed of 22 sequences labelled with various names, for example, *S.vulgaris*, *S.lenis*, *S.tibetica*, *Sidera* sp., *Skeletocutis* sp. and *Schizopora* sp. All samples originated from the Northern Hemisphere (Europe, Asia and North America). In particular, the clade includes all material studied for the first time in the framework the present study (collected from various substrates in Spain, Italy and Greece), as well as sequences from Germany, Estonia, Belarus, Russia, China (incl. Tibet) and the USA (UNITE DOI: SH1262165.09FU).

**Figure 4.**
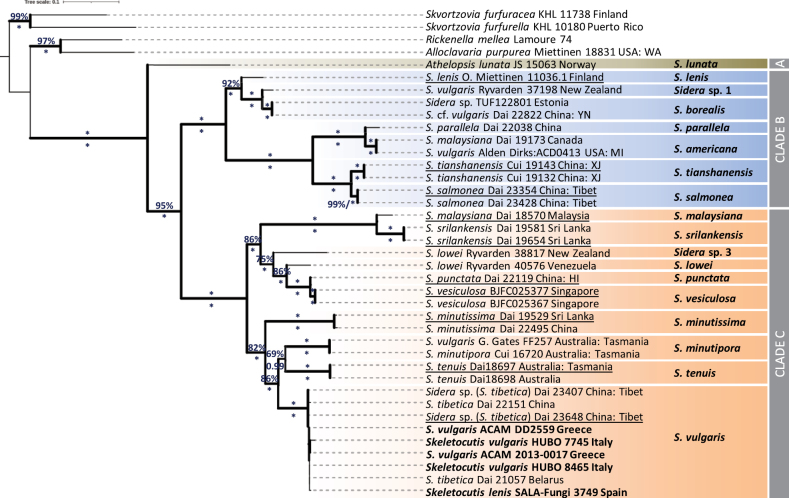
Phylogenetic relationships within the genus *Sidera* inferred by using ML analysis on the concatenated ITS and 28S rDNA sequence dataset. ML BS ≥ 65% and BPP ≥ 0.95 are appended to nodes; asterisk denotes 100% ML BS and/or 1.00 BBP. Specimens studied are followed by their voucher code and geographic origin. Sequences determined in the present study appear in bold, while those representing type material are underlined. The phylogram is rooted with *Skvortzoviafurfuracea* and *Skvortzoviafurfurella*. The scale bar indicates 0.1 expected change per site per branch.

Although represented by fewer sequences, the phylogenetic reconstructions that were based on 28S or on the concatenated ITS and 28S sequences (Suppl. material 2 and Fig. 4, respectively) provided similar topologies as the ITS tree by maintaining the same phylogenetic positions of *S.lunata* and of species within Clades B and C (100%, 1.00). It is also interesting that the unnamed taxa ‘*Sidera* sp. 1’ and ‘*Sidera* sp. 3’ seem to be well-separated from the already known species, as indicated above (Fig. 3). In addition, the specimen MEL:2382752 (Australia), originally identified as *Sidera* sp., appears to be distinct from the two most closely-related taxa, i.e. *S.srilankensis* and *S.malaysiana*; therefore, it is assigned with the provisional name ‘*Sidera* sp. 5’ (Suppl. material 2).

## ﻿Discussion

This study mainly deals with the taxonomic uncertainty associated with collections under the names *S.vulgaris* and *S.lenis*. A great deal of confusion stems from the erroneous initial identifications of such specimens and, as explained below, further obstacles were raised by the description of the (allegedly) new species *S.tibetica* (Liu et al. 2022).

Miettinen and Larsson (2011), who introduced *Sidera* as a new genus to accommodate – amongst others – the dimitic polypores *Sk.vulgaris* and *Sk.lenis*, studied the former by examining three collections: one European (Poland, Niemelä 5981) and two from the Southern Hemisphere. However, sequence data were available only from the latter two. As our results show, the collection from New Zealand (Ryvarden 37198), that was considered to represent *S.vulgaris* and was included as such in several phylogenetic studies, was assigned to clade B in the present study (as ‘*Sidera* sp. 1’). Furthermore, the collection from Tasmania (G. Gates FF257) was grouped in clade C and was identified as *S.minutipora* (Du et al. 2020). Therefore, it is clear that these two collections are not related to *S.vulgaris*. Moreover, several specimens included either as *S.vulgaris* or *S.vulgarissensu lato* in the previous publications (Liu et al. 2021, 2022) were later linked to other species of this genus by the same group of authors (Liu et al. 2023), for example, to *S.americana* (Dai 12730 from USA and Dai 19173 from Canada) and *S.borealis* (Cui 11216 and Dai 22822 from China).

Most importantly, another new *Sidera* species was recently introduced under the name *S.tibetica* (Liu et al. 2022). It was described on the basis of two sequenced specimens from Tibet, but without being examined versus any real/authentic *S.vulgaris* collections. In fact, the three “S.vulgaris” collections included in the aforementioned study were Ryvarden 37198 (New Zealand), Dai 19173 (Canada) and Dai 22822 (China). However, the first of these corresponds to *Sidera* sp. 1 (as the present work demonstrated), the second to *S.americana* and the third to *S.borealis*. In addition, all *S.tibetica* sequences from the work of Liu et al. (2022) [and from other recent publications where this name was also erroneously used (e.g. Volobuev (2023))] were grouped (100%, 1.00) together with a large number of *S.vulgaris* specimens originating from Europe (#15), Asia (#3) and USA (#4). It should additionally be noted that Liu et al. (2021) had indications that the allegedly new species occurred also in Europe (since they have examined the specimen Dai 21057, initially identified as *S.vulgarissensu lato* from Belarus). However, they did not include it in the description of *S.tibetica* (Liu et al. 2022), whereas it was used in their more recent study under this name (Liu et al. 2023). Therefore, it is evident that *S.tibetica* was erroneously introduced as a new species since the respective examined material corresponds to *S.vulgaris*, i.e. the already existing species, previously described from specimens originating from Sweden (Niemelä and Dai 1997). Apparently, the lack of sequence data from the type material of *S.vulgaris* and not including correctly identified collections of the appropriate geographic origin (e.g. Europe) were the main reasons for this major issue detected in the publication of Liu et al. (2022). Hence, *S.tibetica* should be considered as a synonym of *S.vulgaris*.

The molecular evidence provided by the phylogenetic analyses (Fig. 3, Fig. 4 and Suppl. material 2) shows that *S.vulgaris* is well discriminated from *S.lenis* since the respective terminal clades are properly defined and clearly separated. On the other hand, distinguishing *S.vulgaris* from *S.lenis* morphologically remains a difficult task; this resulted in incorrect identifications of the two species in Europe and elsewhere. Until very recently, the distribution of *S.vulgaris* in south Europe was considered to be unknown because, as stated, “… it was easily confused with *S.lenis*” (Bernicchia et al. 2020). It is now clear that *S.vulgaris* is the only species of the genus which is also present in Mediterranean Europe.

Our morphological studies, in conjunction with the verified identity of specimens from DNA sequencing, revealed that the most stable and reliable character to distinguish these two species is the pore size, which is clearly smaller in *S.vulgaris*, on average, more than six pores per mm, as opposed to less than six pores per mm in *S.lenis* (Table 2). The length of the basidia, furthermore, seems to be important in this regard, as they hardly exceed 10 μm in *S.vulgaris*, while they are always longer in *S.lenis*. Regarding the size and shape of basidiospores, our measurements indicate a much wider deviation, particularly with regards to the width, which largely affects the quotient (Q, length/width). Our work also suggests that *S.vulgaris* basidiospores may exceed 1.5 μm in width; this contradicts pertinent generic keys which placed a clear-cut value of 1.5 μm between these species (i.e. Ryvarden and Melo (2017); Bernicchia et al. (2020)). Apparently, this tiny value – and variations thereof – are very difficult to detect with accuracy. In contrast, the average spore length seems to be a more reliable character, since it does not exceed 4 μm in *S.vulgaris*, in contrast to *S.lenis* whose spores are usually longer. In our opinion, the presence of the stellate crystal agglomerations and the mucous deposit on the capitate generative hyphal tips are unstable characters, most likely affected by the age of basidiomata and the microscopy techniques used; thus, they are of questionable taxonomic value. In addition, the most important taxonomic features mentioned in the description of *S.tibetica* (Liu et al. 2022) are similar to (or not differing considerably from) those of *S.vulgaris* (Table 2). The deviations observed in spore size (especially) and pore density are small and they cannot be considered as significant since only three specimens of *S.tibetica* were used for its original description.

**Table 2.** Comparison of key morphological features from collections of *Sideravulgaris* (this work; Niemelä and Dai 1997), *S.tibetica* (Liu et al. 2022) and *S.lenis* (Niemelä and Dai 1997).

	*S.vulgaris* (this work)	*S.vulgaris* (Niemelä and Dai 1997)	*S.tibetica* (Liu et al. 2022)	*S.lenis* (Niemelä and Dai 1997)
**Pores (per mm)**	6–8	6–8	7–8	4–6
**Spores**	3.2–4.0×1.3–1.7 μm, av. 3.64×1.51 μm Q = 2.24–2.60	2.9–3.6×0.9–1.4 μm, av. 3.14×1.08 μm Q = 2.44–3.11	2.9–3.1×1.0–1.1 μm, av. 3.01×1.05 μm Q = 2.78–2.91	3.9–4.9×1.5–2 μm, av. 4.35×1.76 μm Q = 2.29–2.74
**Basidia length**	6.8–11 μm	6.5–8.5 μm	8–9.5 μm	10–13.5 μm
**Skeletals in KOH**	1.7–3.5 μm	2.7–3.5 μm	2.0–4.0 μm	3.5–4.8 μm
**Stellate crystals**	frequent	very rare	frequent	frequent

## ﻿Conclusions

In this work, a large number of *Sidera* sequences (ITS and LSU rDNA) were analysed which included new material from Mediterranean Europe, as well as publicly available sequences. The monophyletic nature of the genus was strongly supported in the generated trees. *Sideralunata* (characterised by a hydnoid hymenophore) was identified as the sister group to the remainder of the genus in the derived phylogeny, while species with poroid hymenophore formed a robustly supported lineage that was subdivided into two major clades. Amongst 23 species in total, five are possibly new to science, but since they are mostly represented by single collections, further work is needed before any definite conclusions could be drawn. The presence of *S.lenis* was assessed in north Europe, Russia and China, while examined collections from south Europe under this name were recovered within *S.vulgaris*. The latter species exhibits a Holarctic distribution. It occurs on dead wood of angiosperms and gymnosperms, including the regions of Eurasia where it was erroneously reported as *S.tibetica*. As we demonstrate, the description of this allegedly new species was based on collections that are hereby identified as *S.vulgaris*. This observation also emphasises the need to proceed with the epitypification of *S.vulgaris* since the type material maintained in Herbarium UPS may be too old for successful sequencing.

## Supplementary Material

XML Treatment for Sidera vulgaris

## ﻿Additional information

### Author ORCIDs

Vassiliki Fryssouli https://orcid.org/0000-0002-8981-6748

Elias Polemis https://orcid.org/0000-0002-2728-7350

Georgios I. Zervakis https://orcid.org/0000-0002-2892-098X

### Data availability

All of the data that support the findings of this study are available in the main text or Supplementary Information.

## Supplementary materials

Supplementary material 1 Detailed characteristics of the phylogenetic analysis performed for each sequence dataset used for the study of *Sidera* collections Data type docx https://binary.pensoft.net/file/1043215This dataset is made available under the Open Database License (http://opendatacommons.org/licenses/odbl/1.0/). The Open Database License (ODbL) is a license agreement intended to allow users to freely share, modify, and use this Dataset while maintaining this same freedom for others, provided that the original source and author(s) are credited.Vassiliki Fryssouli, Elias Polemis, Milton A. Typas, Georgios I. Zervakis

Supplementary material 2 Phylogenetic relationships within the genus *Sidera* inferred by using ML analysis on the 28S rDNA sequence dataset Data type pdf https://binary.pensoft.net/file/1043216This dataset is made available under the Open Database License (http://opendatacommons.org/licenses/odbl/1.0/). The Open Database License (ODbL) is a license agreement intended to allow users to freely share, modify, and use this Dataset while maintaining this same freedom for others, provided that the original source and author(s) are credited.Vassiliki Fryssouli, Elias Polemis, Milton A. Typas, Georgios I. Zervakis
