# Supplementary material for: ﻿Revisiting the phylogeny and taxonomy of the genus Sidera (Hymenochaetales, Basidiomycota) with particular emphasis on S.vulgaris
Source: MycoKeys. 2024 May 7;105:119–37. doi: 10.3897/mycokeys.105.121601 (PMC11094396; doi:10.3897/mycokeys.105.121601)
Supplement: Supplementary material 1 — Detailed characteristics of the phylogenetic analysis performed for each sequence dataset used for the study of Sidera collections [file mycokeys-105-119-s001.docx]

**Supplementary Table S1.** Detailed characteristics of the phylogenetic analysis performed for each sequence dataset used for the study of *Sidera* collections.

|  | ITS | ITS-28S rDNA | 28S rDNA |
| --- | --- | --- | --- |
| No. of *Sidera* sequences | 68 | 35 | 36 |
| Length of alignment | 933 | 1860 | 935 |
| Constant sites | 367 | 1141 | 761 |
| Parsimony-informative sites | 490 | 571 | 121 |
| ML log-likelihood | -8813.952 | -11516.800 | -3227.317 |
| Model substitution (BIC) | SYM+I+G | SYM+I+G | TrN+I+G |
| BI no. of generations | 2,770,000 | 715,000 | 3,220,000 |
| BI 50% credible tree | 2,079 | 28 | 2,416 |
| ML tree presented | Fig. 3 | Fig. 4 | Fig. S1 |

Full names of the used models: SYM+I+G (Symmetrical model with Invariant sites and Gamma distribution); TrN+I+G (Tamura–Nei model with Invariant sites and Gamma distribution).
