# Supplementary material for: ﻿Revisiting the phylogeny and taxonomy of the genus Sidera (Hymenochaetales, Basidiomycota) with particular emphasis on S.vulgaris
Source: MycoKeys. 2024 May 7;105:119–37. doi: 10.3897/mycokeys.105.121601 (PMC11094396; doi:10.3897/mycokeys.105.121601)
Supplement: Supplementary material 2 — Phylogenetic relationships within the genus Sidera inferred by using ML analysis on the 28S rDNA sequence dataset [file mycokeys-105-119-s002.pdf]

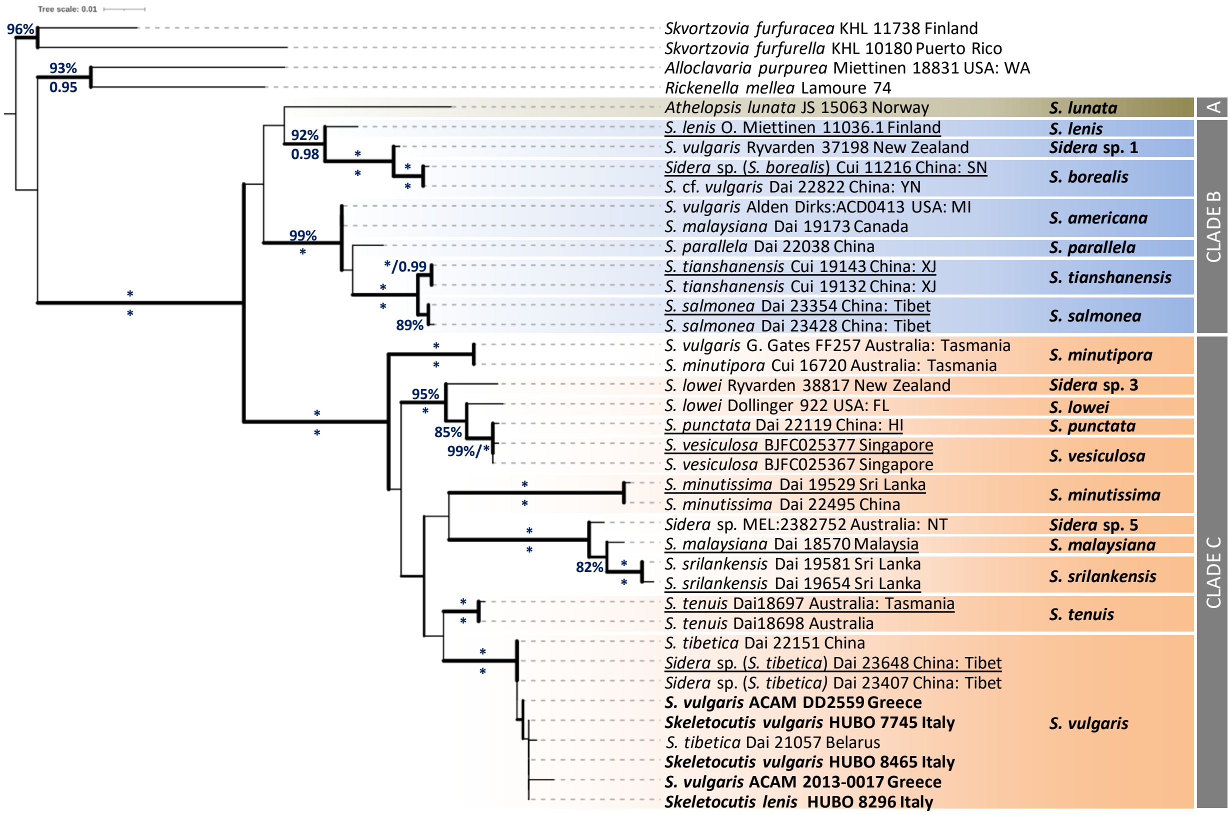

Supplementary Material, Figure S1. Phylogenetic relationships within the genus Sidera inferred by using ML analysis on the 28S rDNA sequence dataset. ML BS ≥ 65% and BPP ≥ 0.95 are appended to nodes; asterisk denotes 100% ML BS and/or 1.00 BBP. Specimens studied are followed by their voucher code and geographic origin. Sequences determined in the present study appear in bold, while those representing type material are underlined. The phylogram is rooted with *Skvortzovia furfuracea* and *Skvortzovia furfurella*. Bar indicates 0.1 expected change per site per branch.
